# Incidence and clinical analysis of asymptomatic intracranial hemorrhage in neonates with cerebral hypoxic-ischaemic risk based on multisequence MR images

**DOI:** 10.1038/s41598-024-62473-6

**Published:** 2024-06-26

**Authors:** Qi Xie, Yan-Hui Liao, Wen-juan He, Gui-Qin Wang

**Affiliations:** 1 Department of Medical Imaging in Nansha, Guangzhou First People’s Hospital, School of Medicine, South China University of Technology, Guangzhou, 511457 China; 2 Department of Nuclear Medicine, Meizhou People’s Hospital, Meizhou, 514031 Guangdong China; 3 Medical Record Department in Nansha, Guangzhou First People’s Hospital, School of Medicine, South China University of Technology, Guangzhou, 511457 China

**Keywords:** Neuroscience, Diseases, Health care, Medical research, Neurology, Risk factors

## Abstract

The incidence and clinical distribution of intracranial haemorrhage (ICH) in neonates at risk of cerebral hypoxia–ischaemia have not been reported in specific studies. Based on conventional magnetic resonance imaging (MRI) versus susceptibility weighted imaging (SWI), this study aimed to analyse the occurrence of asymptomatic ICH in newborns with or without risk of cerebral hypoxia–ischaemia and to accumulate objective data for clinical evaluations of high-risk neonates and corresponding response strategies. 317 newborns were included. MRI revealed that the overall incidence of ICH was 59.31%. The most common subtype was intracranial extracerebral haemorrhage (ICECH) which included subarachnoid haemorrhage (SAH) and subdural haemorrhage (SDH). ICECH accounted for 92.02% of ICH. The positive detection rate of ICECH by SWI was significantly higher than that by T1WI. The incidence of total ICH, ICECH and SAH was greater among children who were delivered vaginally than among those who underwent caesarean delivery. Asymptomatic neonatal ICH may be a common complication of the neonatal birth process, and SWI may improve the detection rate. Transvaginal delivery and a weight greater than 2500 g were associated with a high incidence of ICECH in neonates. The impact of neonatal cerebral hypoxia–ischaemia risk factors on the occurrence of asymptomatic ICH may be negligible.

## Introduction

The aetiology of intracranial haemorrhage (ICH) in newborns is complex and is associated with gestational age, intrauterine distress, birth asphyxia, birth injuries, abnormalities in their own coagulation, abnormal cerebrovascular development, and maternal factors^1–5^. Because the development of the neonatal nervous system is not perfect and because the independent expression ability is weak among infants, the existing clinical evaluation indicators cannot accurately reflect the clinical symptoms and signs of ICH. Therefore, the exact incidence of ICH among neonates is unknown^6^. Some studies have reported that the incidence of symptomatic ICH ranges from 3–4/10,000 to 10–25/10,000^7–9^. With the development of imaging technology, the use of MR cranial imaging in neonates has gradually increased, and several studies have reported that the prevalence of incidentally found asymptomatic ICH in neonates from uncomplicated vaginal deliveries ranges from 6.9% to as high as 63%^10–13^. Carney et al. studied the MR findings of neonates who were clinically judged to be healthy at term and reported that the prevalence of asymptomatic ICH in neonates ranged from 6.9 to 25%^3,13^, with most cases of ICH being subdural. The results from a follow-up MR study and examinations of neurodevelopment from 18 months to 2 years showed no adverse effect on the neurodevelopment of these healthy newborns^3,5^.

The occurrence and clinical distribution of asymptomatic ICH in neonates at risk of hypoxia–ischaemia, which were unexpectedly detected above, have not been reported in specific studies. In contrast, the collection and organization of MR diagnostic and clinical data from these children is of great value for accumulating information and scientific evidence for subsequent follow-up and comparative studies of related developmental conditions.

Therefore, in this paper, we collected clinical and MR data from neonates who underwent cranial MRI in our hospital, compared the detection rate of ICH foci in neonates using conventional MRI sequences and SWI sequences, and evaluated the advantages of SWI sequences in diagnosing ICH. The frequencies of ICH in different clinical risk states were compared to provide objective data for the clinical evaluation of the condition among high-risk neonates and corresponding response strategies.

## Materials and methods

This study employed a retrospective design and the study was approved by the Ethics Committee of Guangzhou First People's Hospital (No. K-2019-166-01). The medical examinations related to this study were all necessary for the diagnosis and treatment of these neonates, and were conducted with the written informed consent of their parents in accordance with the Declaration of Helsinki (revised in 2013).

### Participants

#### Study subjects

A total of 358 hospitalized children who were sent for cranial MRI scans in the neonatal intensive care unit of our hospital between December 2017 and November 2020 were retrospectively analysed. All newborns had complete and detailed medical records. The clinical data collected in this study included the delivery method, gestational age at birth, newborn Apgar score (1 min, 5 min and 10 min), weight at birth, neonatal arterial blood pH value (at birth, during MRI day), and other data collected by medical staff after delivery in the hospital (such as intrauterine infection, asphyxia at birth, etc.). The delivery methods were divided into vaginal delivery, assisted delivery (vacuum or forceps assisted), and caesarean section. The neonatal specialist examination and diagnostic records included pneumonia, pulmonary hyaline membrane disease, blood culture, congenital heart disease (atrial septal defect, ventricular septal defect, patent ductus arteriosus), etc.

A total of 41 infants were excluded because they met at least one of the following criteria: mechanical injuries from forceps (11 patients) or suction (10 patients) via vaginal delivery, an age greater than 28 days (3 patients), the presence of ICH confirmed by a paediatrician with over 5 years of experience in neonatal medical specialties (2 patients), vitamin K deficiency (7 patients), absence of the SWI sequence (22 patients), poor-quality MR images (4 patients), or congenital malformations of the cranial brain on MRI (3 patients). A total of 317 neonates were ultimately included in this study; 111 were preterm infants (gestational age < 37 weeks), and 206 were term infants (gestational age ≥ 37 weeks).

Newborns included in the study received timely symptomatic and supportive treatment based on their condition. According to the evaluation by specialized doctors based on the diagnostic and treatment standards of the Chinese Medical Association for Newborns^14^, the enrolled newborns were not diagnosed with neonatal hypoxic-ischemic encephalopathy.

#### Clinical grouping

Based on the clinical obstetric history and clinical diagnosis, 249 infants with conditions affecting blood oxygen exchange and circulatory function, such as intrauterine infection, history of birth asphyxia, prematurity, neonatal pneumonia, neonatal pulmonary hyaline membrane disease, congenital heart disease (atrial septal defect, ventricular septal defect, patent ductus arteriosus), and sepsis, were included in the high-risk group. The 68 remaining infants who underwent MRI examinations to exclude brain injury due to neonatal hyperbilirubinemia only were included in the low-risk group.

### MR imaging

Patients who were asleep were transferred to the MRI examination room accompanied by ward specialist nursing staff. The head was positioned in the centre of the imaging coil. The parents wore earmuffs and accompanied the child in the examination room to observe whether the child woke up and cried during the examination and to prevent airway obstruction after vomiting.

The following sequences were scanned using a Siemens MR imaging system (Skyra 3.0 T scanner, Germany) with a 20-channel cranial phase-controlled ortho coil (axial planes were positioned parallel to the body of the corpus callosum).

1. T1WI_dark-fluid axial position: repetition time (TR) 1800 ms, echo time (TE) 8.6 ms, slice thickness 4 mm, slice gap 1 mm, flip angle 150°, field of view (FOV) 180 mm × 152 mm, and matrix (256 × 151).
2. T2WI axis position: TR 4000 ms, TE 108 ms, slice thickness 4 mm, slice gap 1 mm, flip angle 150°, FOV 180 mm × 152 mm, and matrix 320 × 189.
3. T2WI_dark-fluid axis position: TR 9000 ms, TE 94 ms, slice thickness 4 mm, slice gap 1 mm, flip angle 150°, FOV 180 mm × 152 mm, and matrix 256 × 173.
4. SWI axis position: TR 27 ms, TE 20 ms, slice thickness 1.5 mm, slice gap 0.3 mm, flip angle 15°, FOV 180 mm × 163 mm, and matrix 256 × 223. The minimum intensity projection (MinIP) map reconstruction thickness 12 mm, and the slice gap 1 mm.

### MR evaluation criteria and data acquisition

The maximum age at the time of examination of the children in this study was 26 days. Therefore, high and slightly high signals on T1WI images were diagnosed as haemorrhagic foci after excluding vascular signals. The diagnostic criteria of Niwa et al. were used for the diagnosis of haemorrhagic foci on SWI^15^: low signals after excluding the possibility of veins on MinIP maps of SW images and mixed high- and low-signal lesions on phase maps were considered haemorrhagic foci. Foci with regular and difficult-to-confirm lesions at the skull base and subcranial region were not included in the statistical analysis. For lesions that were visible on both conventional T1WI sequences and SWI sequences, the extent of the lesions on T1WI and SWI sequences was further compared.

According to the diagnostic criteria listed above, image analysis was independently performed by an experienced radiologist in the field of perinatal MRI who was blinded to the clinical condition of the patient.

The number of neonates with ICH foci at different sites was observed and recorded on the T1WI and SWI sequences. Because distinguishing between micro subarachnoid haemorrhage (SAH) and subdural haemorrhage (SDH) is difficult in neonates, SAH, SDH and epidural haemorrhage (EDH) were categorized as intracranial extracerebral haemorrhage (ICECH). The numbers of neonates with periventricular–intraventricular haemorrhage (PVH-IVH), cerebellar haemorrhage (CH) and intraparenchymal haemorrhage (IPH) were evaluated and recorded. Patients with PVH-IVH, CH and IPH were categorized as having intracranial intracerebral haemorrhage (ICICH).

PVH-IVH is classified into four levels according to the Papile grading system^16^ : Grade I—bleeding only in the germinal stroma; Grade II—Intraventricular haemorrhage without ventricular enlargement; Grade III—Intraventricular haemorrhage with ventricular enlargement; Grade IV—ventricular enlargement accompanied by paraventricular white matter injury or haemorrhagic infarction.

### Statistical analysis

SPSS 25.0 software was used for the statistical analysis. The Pearson χ^2^ test was used for comparison of count data between groups. The measurement data are presented as the means ± standard deviations (${\overline{\text{x}}}$ ± SDs). p < 0.05 was considered significant.

## Results

### Basic clinical data

This study included 167 males and 150 females, 206 full-term infants (272.7 ± 8.1 days) and 111 preterm infants (246.7 ± 8.8 days) with an average age of 8.0 ± 4.8 days (1–26 days) at the time of MR examination. None of the infants had any clinical signs of ICH, such as irritability, poor feeding, vomiting, apnoea, respiratory disturbance, bradycardia, seizures, increased muscle tone or confusion.

The average age on the MRI examination day was 8.2 ± 4.4 days after birth (range 1–22 days) in the preterm infant group, which included 62 patients within 7 days (9 patients within 3 days), 37 patients within 8–14 days, 11 patients within 15–21 days and 1 patient on the 22nd day. The average age on the MRI examination day was 7.8 ± 5.0 days after birth (range 2–26 days) in the full-term group, including 119 patients within 7 days of birth (36 patients within 3 days), 65 patients from 8 to 14 days, 17 patients from 15 to 21 days, and 5 patients from 22 to 26 days.

A total of 249 patients were in the high-risk group, including 129 males and 120 females, with an age of 7.3 ± 4.6 days. 68 patients were in the low-risk group, including 38 males and 30 females, with an age of 10.3 ± 4.8 days (Table 1).

**Table 1:** Basic clinical data of 317 neonates in high-risk group and low-risk group.

Observed indicator	High-risk group (n = 249)	Low-risk group (n = 68)	Statistical analysis	
			χ^2^	*p*
Gender				
Male	129 (51.81%)	38 (55.88%)	0.356	0.585
Female	120 (48.19%)	30 (44.12%)		
Full term or not				
Term	138 (55.42%)	68 (100%)	65.047	0.000
Premature	111 (44.58%)	0		
Mode of delivery				
Vaginal	136 (54.62%)	53 (77.94%)	12.069	0.000
Cesarean	113 (45.38%)	15 (22.06%)		
Complicating disease				
Intrauterine infection	79 (31.73%)	0	25.916	0.000
Birth asphyxia	32 (12.85%)	0 (0%)	9.720	0.002
Neonatal pneumonia	136 (54.62%)	0 (0%)	65.047	0.000
Neonatal pulmonary hyaline membrane disease	7 (2.81%)	0 (0%)	1.955	0.162
Sepsis	22 (8.84%)	0 (0%)	6.456	0.011
Congenital heart disease(right to left shunt)	75 (30.12%)	0 (0%)	12.735	0.000
Apgar score			z	*p*
1 min	8.74 ± 1.733	9.77 ± 0.490	− 4.820	0.000
5 min	9.69 ± 0.808	9.97 ± 0.174	− 3.007	0.003
10 min	9.87 ± 0.433	9.98 ± 0.124	− 2.203	0.028
Gestational age at birth (days)	261.0 ± 15.4	273.1 ± 7.3	− 6.023	0.000
Age at MRI (days)	7.3 ± 4.6	10.3 ± 4.8	− 5.509	0.000
Arterial blood pH			t	*p*
At birth	7.436 ± 0.086	7.500 ± 0.069	5.725	0.000
MRI day	7.522 ± 0.062	7.501 ± 0.058	− 1.740	0.083
Weight at birth (g)	2772.2 ± 547.4	3215.1 ± 404.1	6.222	0.000

Chi square test for counting data (χ^2^*, p*). *T*-test for measurement data with normal distribution (t, *p*); Man-Whitney test for measurement data with non-normal distribution (Z, *p*).

The comparison of clinical data between the high-risk group and the low-risk group is shown in Table 1. The complications of high-risk group of newborns included intrauterine infection, birth asphyxia, neonatal pneumonia, neonatal hyaline membrane disease, sepsis, etc. (Table 1). Blood culture revealed that 22 infants with sepsis were infected with *Staphylococcus epidermidis* (12), *Staphylococcus cepacis* (2), *Streptococcus agalactiae* (3), *Escherichia coli* (3), or *Salmonella group* (2).

### Magnetic resonance detection of ICH in neonates

#### MR manifestations of ICH in neonates

Various types of ICH were detected in a total of 188 patients(59.31%) using conventional T1WI and/or SW, including 173 patients (54.57%) with ICECH, 35 patients (11.04%) with PVH-IVH, 18 patients (5.68%) with intraventricular haemorrhage, 32 patients (10.09%) with CH, and 30 patients (9.46%) with IPH.

**Figure 1:**
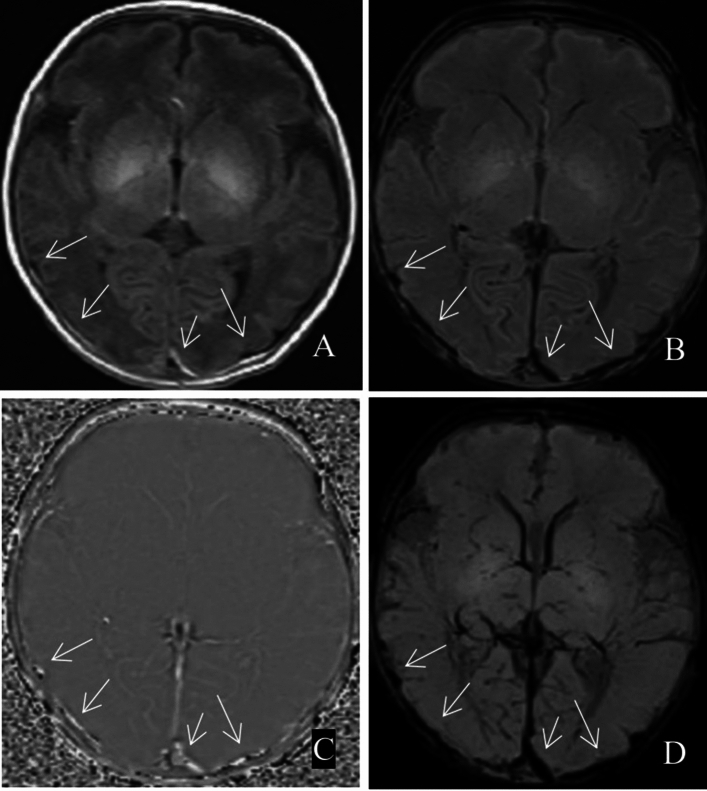
Bilateral parietooccipital subdural haemorrhage (white arrow). (**A**) T1WI; (**B**) SWI; (**C**) phase map; (**D**) MinIP map.

**Figure 2:**
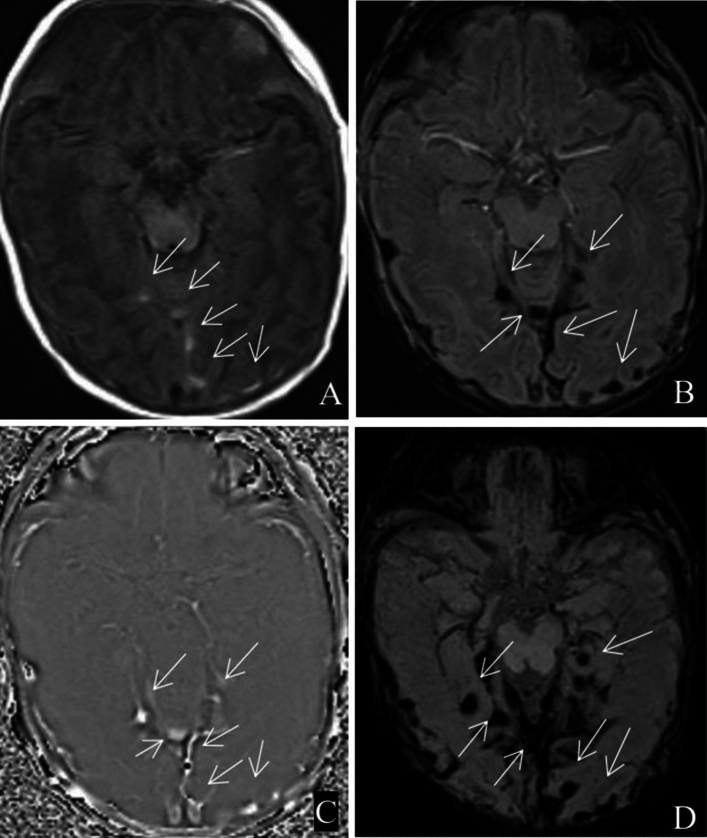
Scattered subarachnoid haemorrhage in tentorium cerebellum and occipital sulcus (white arrow). (**A**) T1WI; (**B**) SWI; (**C**) phase map; (**D**) MinIP map.

Among the 173 patients with ICECH, 172 had SAH (combined with SDH in 7 patients) and only 1 had SDH without SAH. No cases of EDH were detected. SAH can be secondary to SDH, leading to the coexistence of both. The differentiation of these conditions on MRI is sometimes difficult. SDH manifests mainly as a crescent-shaped abnormal signal under the internal plate of the skull. The haemorrhage signal was shown as a high signal on T1WI, a low signal on SWI in the form of stripes, a mixed high and low signal on the phase diagram and a low signal on the MinIP map. The display range on the MinIP diagram of the SWI sequence has an obvious amplification effect (Fig. 1). SAH is common in neonates with ICH, and haemorrhagic foci in the cerebral sulci and pools are easily distinguished from SDH (Fig. 2).

**Figure 3:**
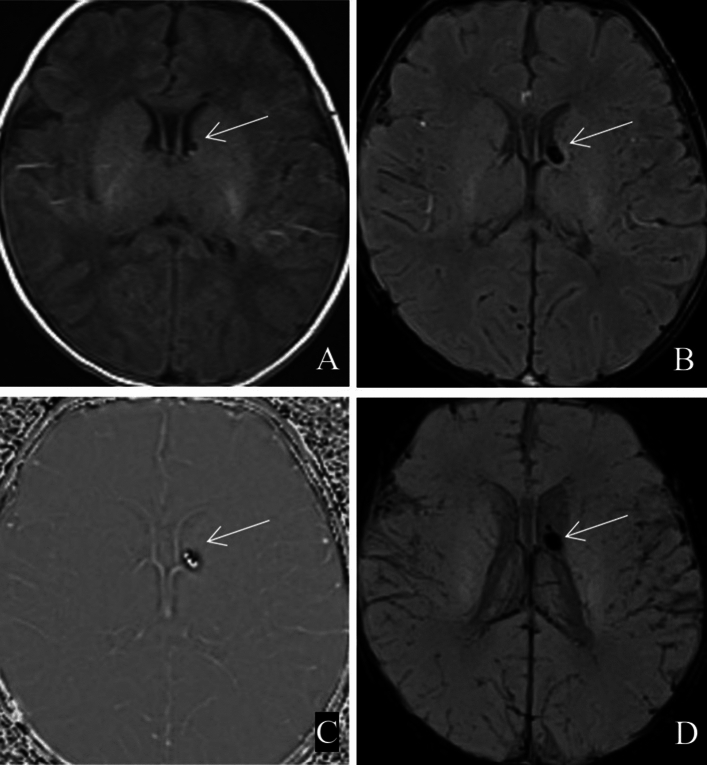
Subependymal haemorrhage near the anterior horn of the left lateral ventricle(white arrow). (**A**) T1WI; (**B**) SWI; (**C**) phase map; (**D**) MinIP map.

**Figure 4:**
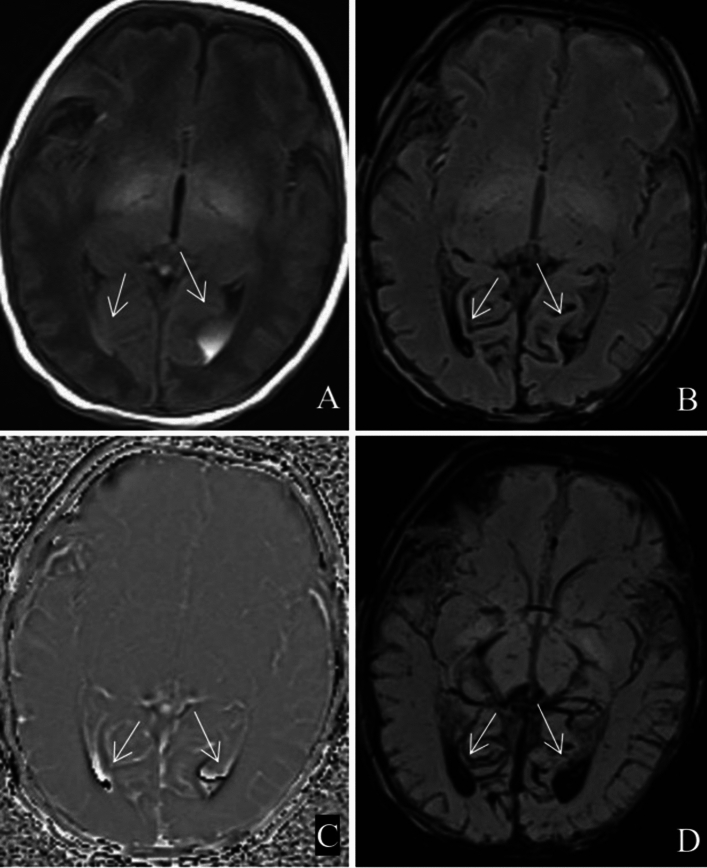
Haemorrhage in occipital horn of bilateral lateral ventricles (white arrow). (**A**) T1WI; (**B**) SWI; (**C**) phase map; (**D**) MinIP map.

In 35 patients with PVH-IVH, the haemorrhage was confined to the subependyma or the germinal stroma (e.g., Fig. 3) or penetrated the ependyma to cause intraventricular haemorrhage (e.g., Fig. 4); therefore, these patients were collectively referred to as having PVH-IVH. None of these 35 children with PVH-IVH had corresponding ventricular dilatation.

**Figure 5:**
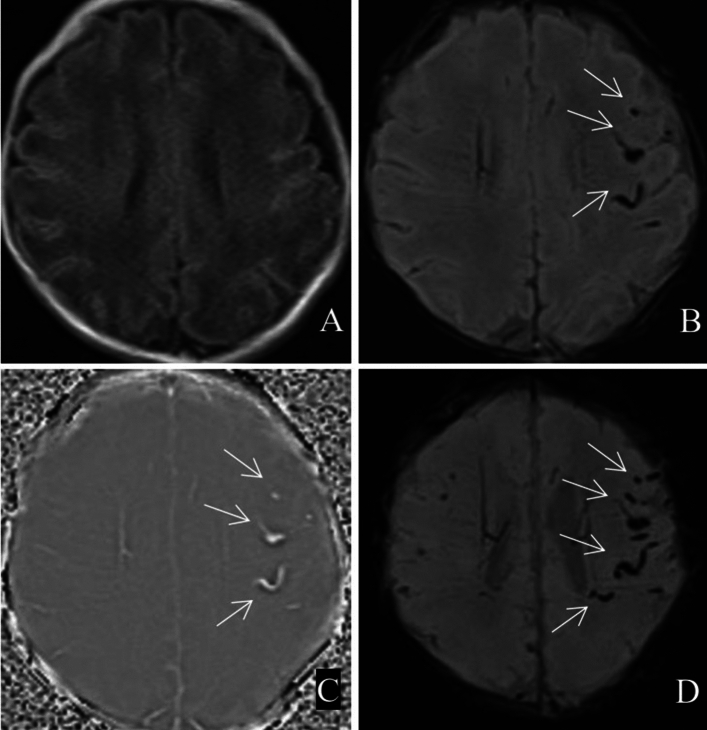
The left frontoparietal vein is tortuous with haemorrhage (white arrow). (**A**) T1WI; (**B**) SWI; (**C**) phase map; (**D**) MinIP map.

ICH in the brain parenchyma other than the periventricular germinal matrix was rare, and only 9 cases were detected in this study. Most of them were caused by increased pressure and rupture of the capillary bed after obstruction of the terminal small venous vessels, which were visible as tortuous venous vessels (Fig. 5).

**Figure 6:**
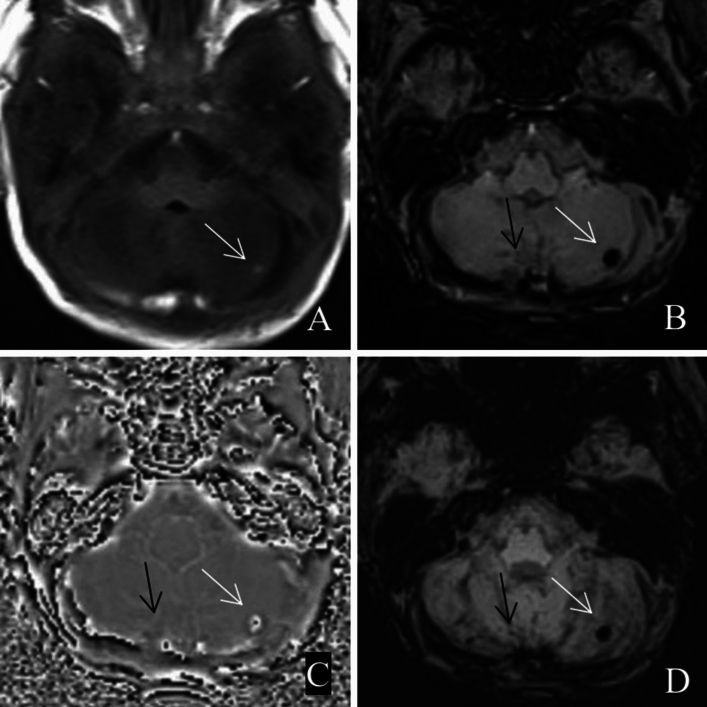
Left cerebellar hemisphere haemorrhage (white arrow); Suspicious hemorrhage in the right cerebellar hemisphere (black arrow). (**A**) T1WI; (**B**) SWI; (**C**) phase map; (**D**) MinIP map.

In 32 patients with ICH, speckled and round-like haemorrhagic foci were mostly observed and were located in the cerebellar cortex. Therefore, distinguishing from secondary SAH that spread to the cerebellar surface with cerebrospinal fluid is sometimes difficult (Fig. 6).

#### Comparison of conventional T1WI sequences and SWI sequences for the detection of ICECH in neonates

A total of 133 cases of different degrees of ICECH were detected using T1WI, and 173 cases were detected using SWI. A significant difference was observed between the rate of positive detection of ICECH foci in neonates using T1WI and SWI (χ^2^ = 190.73, p < 0.001) (Table 2). Moreover, SWI MinIP images were able to detect a greater number of ICH foci and to show a clearer and wider range of lesions than conventional T1WI scans (e.g., Fig. 2).

**Table 2:** Detection rate of IEH in neonates by T1WI and SWI, n(%).

Groups	Detection	No detection	χ^2^	*p*
T1WI	133 (76.9)	40 (23.1)	190.73	< 0.001
SWI	173 (100)	0 (0)		

Pearson χ^2^ test; *IEH* intracranial extracerebral haemorrhage.

### Clinical analysis of ICH in neonates

Table 3 shows the distribution of 188 newborns with ICH by sex, term, mode of birth, birth weight, and risk group.

**Table 3:** Clinical distribution of ICH in 317 neonates, n (%).

Characteristic	Variable	With ICH (n = 188)	Without ICH (n = 129)	χ^2^	*p*
Gender	Male	103 (61.68)	64 (38.32)	0.822	0.365
	Female	85 (56.67)	65 (43.33)		
Full term or not	Term neonates	122 (59.22)	84 (40.78)	0.002	0.967
	Premature neonates	66 (57.66)	45 (40.54)		
Mode of delivery	vaginal	135 (71.43)	54 (28.57)	28.502	0.000
	Cesarean	53 (41.40)	75 (58.6)		
Weight at birth	< 2500 g	40 (50.63)	39 (49.37)	3.280	0.070
	≥ 2500 g	148 (62.18)	90 (37.82)		
Risk grouping	High-risk groups	150 (60.24)	99 (39.76)	0.420	0.517
	Low-risk groups	38 (55.88)	30 (44.12)		

Pearson χ^2^ test; *ICH* intracranial heamorrhage.

The overall incidence of ICH among the 317 children was 59.31% (188/317). As shown in Table 3, the incidence of ICH in each of these groups was as follows: 61.68% (103/167) in males and 56.67% (85/150) in females; 57.66% (64/111) in preterm infants and 59.22% (122/206) in term infants; 71.43% (135/189) in infants born by vaginal delivery and 41.41% (53/128) in infants born by caesarean delivery; 50.63% (40/79) in infants with a weight at birth < 2500 g and 62.18% (148/238) in infants with a weight at birth ≥ 2500 g; and 60.24% (150/249) in the high-risk group and 55.88% (38/68) in the low-risk group. The incidence of ICH in newborns born through vaginal delivery was significantly higher than that in newborns born through caesarean delivery. The incidence of ICH in newborns with a birth weight greater than or equal to 2500 g was 11.55% higher than that in newborns with a birth weight less than 2500 g, although the difference was not statistically significant.

Thirty-five infants were diagnosed with PVH-IVH, including 17 with Papile grade I and 18 with grade II. The incidence of PVH-IVH was not significantly higher in preterm infants than in term infants (χ^2^ = 1.063, p = 0.303). Additionally, the differences between the term and preterm groups for ICH (χ^2^ = 0.125, p = 0.723) and intracranial extracerebral haemorrhage (χ^2^ = 0.139, p = 0.709) were not significant (Table 3).

Thirty-one neonates with PVH-IVH were in the high-risk group. In the high-risk group, the incidence of PVH-IVH in premature infants was significantly higher than that in full-term infants (Table 4). The incidence of neonatal pulmonary hyaline membrane disease in premature infants was significantly higher than that in full-term infants (Table 4). Except for congenital heart disease, the incidence of other complicating diseases was higher in term infants; in particular, the incidence of birth asphyxia in term newborns was a significantly higher than that in premature infants (Table 4).

**Table 4:** Incidence of ICECH, PVH-IVH and Complicating disease in term and premature neonates of high risk group, n (%).

High risk group		Term (n = 138)	Premature (n = 111)	χ^2^	*p*
ICECH	Yes	79 (57.2)	59 (53.15)	0.417	0.518
	No	59 (42.8)	52 (46.85)		
PVH-IVH	Yes	9 (6.5)	22 (19.8)	9.981	0.002
	No	129 (93.5)	89 (80.2)		
Intrauterine infection	Yes	45 (32.61)	34 (30.63)	0.111	0.739
	No	93 (67.39)	77 (69.34)		
Birth asphyxia	Yes	28 (20.29)	4 (3.6)	15.294	0.000
	No	110 (79.71)	107 (96.4)		
Neonatal pneumonia	Yes	81 (58.7)	55 (49.55)	2.076	0.150
	No	57 (41.3)	56 (50.45)		
Neonatal pulmonary hyaline membrane disease	Yes	0 (0)	7 (6.31)	8.954	0.003
	No	138 (100)	104 (93.69)		
Sepsis	Yes	16 (11.59)	6 (5.41)	2.925	0.087
	No	122 (88.41)	105 (94.59)		
Congenital heart disease(Right to left shunt)	Yes	36 (26.09)	39 (35.14)	2.041	0.161
	No	102 (73.91)	72 (64.86)		

Pearson χ^2^ test; *ICECH* intracranial extracerebral haemorrhage, *PVH-IVH* periventricular–intraventricular haemorrhage.

The incidence of total ICH, ICECH and SAH was significantly higher among children who were delivered vaginally than among those who were delivered by caesarean (Table 5). In contrast, no significant difference in the incidence of any of the different types of intracerebral haemorrhage was observed between the transvaginal delivery group and the caesarean delivery group (Table 5).

**Table 5:** Incidence of intracranial hemorrhage in 317 neonates by mode of delivery, n(%).

Type		Vaginal (n = 189)	Cesarean (n = 128)	χ^2^	*p*
ICH	Yes	135 (71.43)	53 (41.41)	28.502	0.000
	No	54 (28.57)	75 (58.59)		
ICECH	Yes	87 (46.03)	38 (29.69)	8.536	0.003
	No	102 (53.97)	90 (70.31)		
PVH-IVH	Yes	21 (11.11)	14 (10.94)	0.002	0.961
	No	168 (88.89)	114 (89.06)		
IPH	Yes	15 (7.94)	15 (11.72)	1.274	0.259
	No	174 (92.06)	113 (88.28)		
CH	Yes	15 (7.94)	17 (13.28)	2.402	0.121
	No	174 (92.06)	111 (86.72)		
SAH	Yes	127 (67.20)	46 (35.94)	30.089	0.000
	No	62 (32.80)	82 (64.06)		

Pearson χ^2^ test; *ICH* intracranial haemorrhage, *ICECH* intracranial extracerebral haemorrhage, *PVH-IVH* periventricular–intraventricular haemorrhage, *IPH* intraparenchymal haemorrhage, *CH* cerebellar haemorrhage, *SAH* subarachnoid haemorrhage.

The sample of 317 patients included 0 infants weighing less than 1500 g, 79 low-birthweight infants (less than 2500 g), 235 normal-birthweight infants (2500–4000 g), and 3 high-birthweight infants (weight greater than 4000 g). The incidence of ICH among non-low-birthweight infants was greater than that among low- birthweight infants, and the difference was significant (Table 6). The rate of transvaginal delivery was 63.4% (151/238) in the non-low-birthweight infant group, which was significantly higher than that in the low- birthweight infant group (48.1%, 38/79).

**Table 6:** Incidence of intracranial haemorrhage by weight at birth in 317 neonates, n(%).

Type		< 2500 g (n = 79)	≥ 2500 g (n = 238)	χ^2^	*p*
ICH	Yes	40 (50.63)	148 (62.18)	3.280	0.070
	No	39 (49.37)	90 (37.82)		
ICECH	Yes	33 (41.77)	140 (58.82)	6.956	0.008
	No	46 (58.23)	98 (41.18)		
PVH-IVH	Yes	11 (13.92)	24 (10.08)	0.890	0.345
	No	68 (86.08)	214 (89.92)		
IPH	Yes	8 (10.13)	22 (9.24)	0.054	0.816
	No	71 (89.87)	216 (90.76)		
CH	Yes	11 (13.92)	21 (8.82)	1.700	0.192
	No	68 (86.08)	217 (91.18)		
SAH	Yes	33 (41.77)	139 (58.40)	6.610	0.010
	No	46 (58.23)	99 (41.60)		

Pearson χ^2^ test; *ICH* intracranial haemorrhage, *IEH* intracranial extracerebral haemorrhage, *PVH-IVH* periventricular-intraventricular haemorrhage, *IPH* intraparenchymal haemorrhage, *CH* cerebellar haemorrhage, *SAH* subarachnoid hemorrhage.

## Discussion

### Clinical presentation, aetiology–pathology and magnetic resonance detection of ICH in neonates

Before imaging technology was widely used in neonatal cranial examination, neonatal ICH (NICH) was diagnosed based mainly on clinical manifestations and lumbar puncture of the spinal fluid. Neonates clinically diagnosed with intracranial haemorrhage present with irritability, poor feeding, vomiting, apnoea, respiratory disorders, bradycardia, seizures or confusion and have a poor prognosis^17,18^. Therefore, NICH is regarded as a serious disease of neonates^1,2^.

The current causes of ICH in neonates are thought to be birth injury, hypoxia, coagulation dysfunction, and abnormal cranial vascular development^1,2,11,12^. Depending on the location, several types of ICH^6,19^ have been defined, including PVH-IVH, also known as germinal matrix intraventricular haemorrhage (GM-IVH), SDH, IPH, CH and primary SAH. PVH-IVH/GM-IVH is most common among premature infants, and SDH is the most common obstetric ICH. IPH occurs mostly in term infants and is caused by venous infarction following small vein embolism. SAH is a very common ICH in neonates^6,19^.

The widespread use of MRI for neonatal cranial examinations has revealed the high incidence of asymptomatic ICH among healthy neonates without risk factors for NICH, referred to as silent NICH^11,12^. Based on MR images, a review of the literature revealed that the incidence of asymptomatic ICH in healthy newborns differed significantly among studies (6.9–63%)^10–13,20^. On MR images, different signals generated by different magnetic susceptibilities of haemoglobin metabolites in the haemorrhagic focus are the basis for judging haemorrhage. ICH absorption may occur from the first day to the fifth week after birth^3^. Rooks et al. concluded that most SDHs occurring at birth can be absorbed within 1 month^10^. Therefore, the age at MRI examination is an important factor determining the incidence of SDH among neonates.

At present, conventional T1WI, T2WI and SWI sequences are used to diagnose ICH. The conventional sequence is greatly affected by the changes in the haemoglobin metabolite magnetization effect in the haemorrhage focus, which makes it difficult to identify the micro haemorrhage focus and the haemorrhage with a small difference in signals between the haemorrhage focus and the surrounding structure. The above factors increase the difficulty of diagnosing ICH with conventional sequences^21^.

SWI is a 3D gradient echo imaging technology that uses the difference in magnetic sensitivity between different tissues to improve the contrast between tissues and is extremely sensitive to susceptibility changes. SWI has the characteristics and advantages of thin layers, three dimensions, high resolution and a high signal-to-noise ratio, which can significantly reduce the partial volume effect on imaging. The phase diagram of SWI shows that extremely fine differences in magnetic susceptibility lead to changes in the magnetic field, which is highly sensitive to the microhaemorrhage. The MinP image can provide a high-resolution image of the cerebral vein structure by displaying the low signal in the image with a certain thickness through reconstruction to better display the continuity of the vein vessel. SWI has obvious advantages in differentiating the continuous low signal of venous vessels from the discontinuous low signal of bleeding foci^14,22^.

Previous studies have shown that SWI sequences are significantly superior to conventional MRI sequences in terms of the detection rate and display clarity of the cerebral parenchyma and intraventricular haemorrhagic lesions in neonates, especially third ventricle, midbrain aqueduct and fourth ventricle haemorrhage in children with subependymal intraventricular haemorrhage^23,24^. However, the detection rate of SWI in ICECH, such as subdural haemorrhage and subarachnoid haemorrhage, is not significantly better than that of conventional MRI sequences.

In this study, 173 cases of ICECH were detected using SWI, and only 133 cases were detected using T1WI. The haemorrhage foci detected in the T1WI sequence were all visible in the SWI sequence. The detection rate of SWI was significantly higher than that of the conventional T1WI sequence (p < 0.001), indicating that SWI is more sensitive for detecting ICECH foci than conventional T1WI. Moreover, SWI, especially MinIP mapping, revealed clearer and more extensive lesions.

Moreover, we analysed 40 cases of ICECH that could be detected only on SWI but not on T1WI, which suggested that most of these lesions were located in the cerebellar curtain and longitudinal fissure pool and that most of them were small lesions. The reasons for the high sensitivity of SWI for detecting such small haemorrhagic foci include the following: (1) The slice thickness of conventional T1WI sequences is larger, mostly 4–5 mm. Thus, small lesions can easily be missed or made unrecognizable by T1WI due to the partial volume effect. However, SWI covers a thin layer with high spatial resolution, and a slice thickness of 1.5 mm is routinely set in our hospital to better visualize small lesions and reduce the partial volume effect. (2) The magnetic field perturbation generated by paramagnetic material is larger than its own volume, which also suggests that the area of the haemorrhagic foci shown on SWI is larger than the actual size. According to the literature^25,26^, the magnification of haemorrhagic foci on SWI MinIP maps varies at different times after haemorrhage and can be increased by an average of 2–5 times, thus improving the sensitivity of detecting haemorrhagic foci. (3) The signal of the haemorrhagic foci on the T1WI sequence is more complex, and the signals caused by changes in the nature of the haemorrhagic material over time also show different characteristics that can be greater than, equal or less than the signal of the surrounding brain parenchyma. Thus, the T1WI sequence may lead to a false negative diagnosis. In contrast, the imaging characteristics of SWI indicate that the detection of paramagnetic substances produced by haemoglobin degradation in the haemorrhage focus with SWI is stable for a long time after the haemorrhage, which extends the diagnostic window^27^.

The above results also suggest that SWI has a higher positive detection rate for intracerebral microbleeds than conventional T1WI sequences and can be an effective supplement to conventional sequences. If a clinical suspicion of ICH in the newborn or suspected haemorrhagic focus is detected on conventional T1WI, a SWI examination should be subsequently performed. The advantages of SWI in the diagnosis of haemorrhagic lesions, especially in the detection of microbleeds, include the ability to provide effective information for assisting in the clinical diagnosis, guiding clinical treatment and evaluating the prognosis through the early and efficient detection of intracranial haemorrhage.

### The incidence and distribution of ICH in neonates at risk of cerebral hypoxic ischaemia

This observational study was conducted on neonates affected by risk factors for hypoxic–ischaemic encephalopathy. Based on the clinical obstetric history and clinical diagnosis, factors such as birth injuries, haemorrhagic diseases of the haematological system, and cerebrovascular malformations were excluded. Neonates younger than 28 days of age with factors affecting blood oxygen exchange and circulatory function were included in the high-risk group, and these children suffered from one or more of the following diseases: intrauterine infection, birth asphyxia history, prematurity, neonatal pneumonia, neonatal pulmonary hyaline membrane disease, or sepsis. Infants who underwent MRI to exclude brain injury due to neonatal jaundice were included only in the low-risk group (68 patients).

Among the 317 newborns evaluated in this study, ICH was detected using SWI in 188 newborns, and none of them had clinical manifestations of ICH, which is an unexpected finding of asymptomatic ICH. The overall incidence of ICH was 59.31% (188/317). The incidence of ICH in each group was 61.68% among males and 56.67% among females, 57.66% among preterm infants and 59.22% among term infants, and 60.24% in the high-risk group and 55.88% in the low-risk group. The difference in the overall incidence of ICH within each group was not significant.

We also found that asymptomatic ICH in neonates occurred at multiple sites in both the high- and low-risk groups, consistent with the findings of Carney O et al., but that haemorrhage in the cerebellum was uncommon^3^. The most common subtype was ICECH (SAH + SDH), with 173 cases and an incidence of 54.57%, accounting for 92.02% of intracranial haemorrhages. Our results are similar to those from the prospective study by Rooks et al., which reported a 46% incidence of SDH among 101 asymptomatic term neonates (scanned within 3 days of birth using a 1.5 T MRI scanner)^10^, but higher than those from the study by Carney et al. (2007–2021), who also used 3 T MRI and performed the scan and 26–27% of SDH cases were detected in neonates delivered by vaginal suction at 1 to 5 weeks of age^3,12^. The lower detection rates in their studies may be related to the use of conventional T1WI and T2WI sequences only.

Several previous studies have shown^28–30^ that PVH-IVH, which includes mainly periventricular germinal matrix haemorrhage, lateral ventricular haemorrhage and choroid plexus haemorrhage, is a common type of ICH in preterm infants and is particularly prevalent in preterm infants below 32 weeks of gestational age with low birth weight. Various prenatal, perinatal and postnatal factors have been identified as independent risk factors for GM-IVH in preterm infants. These factors include in vitro fertilization, lack of prenatal care, maternal prenatal steroid deficiency, chorioamnionitis, multiple pregnancies, HIV exposure, foetal distress, vaginal delivery, delivery status, male sex, lower gestational age and birth weight, resuscitation at birth, intubation in the delivery room, anaemia (low haematocrit), and blood transfusion^31–39^. Other risk factors include clinically significant ductus arteriosus^40^, pneumothorax^30,41^, a high inhaled oxygen concentration (FiO_2_) in the first 24 h, early and late sepsis^31^, postpartum hydrocortisone therapy for hypotension, the use of inotropic medications^32,38,41,42^, respiratory distress syndrome requiring mechanical ventilation, hyponatremia, hyperglycaemia^33^, hypercarbonic acidosis^33,35^ and severe metabolic acidosis^32,43^. Studies have also shown that preterm infants born in lower quality health care facilities are more likely to develop GM-IVH^32^. Equally important genetic risk factors include factor V Leiden (Arg506Gln), prothrombin (G20210A) gene mutations, and methylenetetrahydrofolate reductase (MTHFR 1298A>C) polymorphisms^44–46^.

Further analysis of the cases in this study revealed that the incidence of PVH-IVH was significantly higher in preterm infants than in term infants under the influence of hypoxic-ischaemic risk factors in children. This result suggested that comorbidities associated with the hypoxic-ischaemic risk are more likely to lead to periventricular germinal matrix haemorrhage in premature infants. The high-risk newborns in this study received timely symptomatic and supportive treatment according to their condition, and no infants were diagnosed with HIE. In addition, our data suggest that weight and the route of delivery do not influence the incidence of PVH–IVH.

According to the literature, local mechanical trauma, head extrusion and the overlap of cranial sutures during vaginal delivery lead to vein compression and the rupture of bridging veins and capillaries, which is a possible mechanism of SDH^6,47–49^. Newborn SDH is particularly common near dural folds bearing venous sinuses, which supports the role of the venous plexus in SDH^50^. Ami et al. used magnetic resonance imaging during childbirth to reveal the migration of cerebrospinal fluid from the ventricular and pericerebral spaces to the posterior fossa as well as global folding of the cerebral mass when the foetal head passes through the vagina^51^. This result suggests that the increased pressure in the posterior cranial fossa compresses the foramen magnum, which can lead to the obstruction of intracranial venous reflux and increase the risk of venous bleeding. Thus, compared with caesarean section, vaginal delivery inevitably compresses the neonatal head and increases the risk of ICH.

A study by Nikam et al. revealed a significantly higher incidence of delivery-related SDH in the vaginal delivery group than in the caesarean delivery group^6^. Our study corroborates this phenomenon: the incidences of total ICH, ICECH and SAH were significantly higher in the group of children delivered vaginally than in the group of children delivered by caesarean section. Our observations also revealed that the incidences of ICECH and SAH were higher in the group of children weighing more than 2500 g than in those weighing less than 2500 g (significant difference). Further analysis revealed a higher rate of vaginal delivery in the group of children weighing more than 2500 g than in those weighing less than 2500 g, suggesting that the difference in ICH due to weight factors may originate from the influence of the delivery route.

The present study has many limitations. The study was a retrospective analysis, with no completely healthy newborns in the control group. Children who were hospitalized only for neonatal jaundice in the database were used as the control group. In the diagnosis of neonatal ICH lesions, especially SAH and SDH, the diagnostic standard was derived from the literature and clinical experience because pathological findings were not available, and a “gold standard” was not available to verify the diagnostic results of MRI. The age at MRI examination ranged from 1 to 26 days, which could have a certain impact on the rate of ICH detection. No follow-up data were included in the analysis, and no definite conclusions can be drawn regarding the clinical significance of ICH in neonates at risk of hypoxia–ischaemia. Further follow-up studies on the neurodevelopmental status of children are needed to clarify the clinical significance of hypoxic–ischaemic risk factors for neonates with asymptomatic intracranial haemorrhage.

The results of this study and relevant literature reports suggest that asymptomatic NICH is a common complication of neonatal birth and that SWI MR sequences may improve the detection rate. Transvaginal delivery and a weight greater than 2500 g are risk factors for ICECH in neonates. Comorbidities associated with the hypoxic-ischaemic risk are more likely to lead to periventricular germinal matrix haemorrhage in premature neonates. This study also suggested that the incidence of asymptomatic NICH among children with risk factors for hypoxia ischaemia does not differ significantly from the incidence among children without an associated risk.

## Data Availability

The data that support the findings of this study are available from correspondence author but restrictions apply to the availability of these data, which were used under licence for the current study, and so are not publicly available.
